# Carbapenemase-producing *Pseudomonas aeruginosa* from central Greece: molecular epidemiology and genetic analysis of class I integrons

**DOI:** 10.1186/1471-2334-13-505

**Published:** 2013-10-29

**Authors:** Apostolos Liakopoulos, Angeliki Mavroidi, Efstathios A Katsifas, Alexandros Theodosiou, Amalia D Karagouni, Vivi Miriagou, Efthymia Petinaki

**Affiliations:** 1 Department of Microbiology, University Hospital of Larissa, Larissa, Greece; 2 Faculty of Biology, Microbiology Group, University of Athens, Athens, Greece; 3 Department of Urology, University Hospital of Larissa, Larissa, Greece; 4 Laboratory of Bacteriology, Hellenic Pasteur Institute, Athens, Greece; 5 Department of Microbiology, Medical School, University of Thessaly, Biopolis, Larissa, Greece

**Keywords:** *Pseudomonas aeruginosa*, MDR, Carbapenems, VIM, Integrons, MLST

## Abstract

**Background:**

Multidrug-resistant *Pseudomonas aeruginosa* is a serious challenge for antimicrobial therapy of nosocomial infections, as it possesses several mechanisms of antimicrobial resistance. In Central Greece, a sudden increase of infections caused by carbapenem-resistant *P. aeruginosa* was observed during 2011, indicating the need for further analysis.

**Methods:**

Five-hundred and sixty-eight *P. aeruginosa* isolates were collected consecutively during an 8-month period in 2011 from inpatients treated in three hospitals in the Thessaly region (1,000,000 habitants) of Greece. Carbapenem-resistant *P. aeruginosa* (n = 284) were characterized by antimicrobial susceptibility testing and β-lactamase content, and the genetic relatedness of carbapenemase-producing isolates was assessed by BOX-PCR, multilocus sequence typing, and eBURST analysis. Mapping of the class I integrons of Verona integron-encoded metallo-β-lactamase (VIM)-carrying isolates was also performed, and clinical data of the VIM producers were reviewed.

**Results:**

Eighty (14.1%) out of the 568 *P. aeruginosa* isolates recovered from clinical specimens were VIM producers. Multilocus sequence typing revealed high prevalence of the international clones ST111 and ST235 among *bla*VIM-2- and *bla*VIM-4-positive isolates, respectively. *bla*VIM-17 was identified in an isolate of a novel sequence type (ST1457). *bla*_VIM_ gene cassettes were carried by five distinct class I integrons, including two novel ones.

**Conclusions:**

Since the first report of VIM-producing *P. aeruginosa* in 2000, this microorganism still remains among the most prevalent multidrug resistant pathogens in Greece. The spread of VIM-producers belonging to the most common international clones (ST111 and ST235), the spread of integrons of divergent structures, and the emergence of novel integrons underscore their ongoing evolution.

## Background

*P. aeruginosa* is a leading cause of nosocomial infections, especially in immunocompromised patients. The high prevalence of multidrug-resistant (MDR) *P. aeruginosa* is a serious challenge for antimicrobial therapy. MDR *P. aeruginosa* possesses several mechanisms of antimicrobial resistance; over-expression of the intrinsic AmpC-type cephalosporinase, which confers resistance to ceftazidime, inactivation or down-regulation of the OprD porin, conferring resistance to carbapenems, and upregulation of the MexAB-OprM or other efflux pumps of the resistance-nodulation-cell division family, which can also confer resistance to carbapenems, as well as to quinolones, and in some cases, to aminoglycosides [1].

Acquired resistance to β-lactams is often reported in *P. aeruginosa* as a result of the acquisition of extended spectrum β-lactamases (ESBLs), such as the PER-1, VEB-1, TEM, and SHV type ESBLs [2]. Acquired resistance to carbapenems can also be mediated by the production of either specific carbapenem-hydrolyzing β-lactamases (carbapenemases), such as the metallo-β-lactamases (MBLs), including the Verona integron-encoded β-lactamase (VIM) and imipenemase (IMP), or by *Klebsiella pneumoniae* carbapenemase (KPC) enzymes and GES/IBC-like enzymes [3-5]. MBLs hydrolyze almost all clinically-available β-lactams except monobactams, and the respective *bla* genes are often carried on transferable structures, known as integrons [4]. Nosocomial outbreaks caused by MBL-producing *P. aeruginosa* have been reported in several countries worldwide [6-10]. Implementation of multilocus sequence typing (MLST) has facilitated the elucidation of the global epidemiology and evolution of these multidrug-resistant pathogens [11-14].

In the prefecture of Thessaly (Central Greece), the rate of isolation of carbapenem-resistant *P. aeruginosa* was around 33% during the period 2007–2010, but a sudden increase to 50% was observed in 2011 (E. Petinaki, unpublished data). For this reason, the molecular epidemiology of carbapenemase-producing *P. aeruginosa* isolated from Thessaly was investigated. The genetic context of the *bla*_VIM_ genes was also studied.

## Methods

### Bacterial isolates and antimicrobial susceptibility testing

Five-hundred and sixty-eight *P. aeruginosa* isolates were collected from March to October 2011 from the same number of inpatients treated at three hospitals in Thessaly, Greece. Three hundred and fifty isolates were recovered from the University Hospital of Larissa (UHL), a 600-bed tertiary care hospital, while the remaining isolates were collected from the general hospitals of Volos and Trikala. Species identification and antimicrobial susceptibility testing were performed using the Vitek-2 automated system (BioMerieux Inc., Marcy l′ Etoile, France), according to the interpretive criteria of the Clinical and Laboratory Standards Institute [15]. MDR *P. aeruginosa* are generally defined as *P. aeruginosa* resistant to imipenem, ciprofloxacin, and amikacin (minimum inhibitory concentrations (MICs) of ≥16 mg/L, ≥4 mg/L, and ≥64 mg/L, respectively). ESBL production was tested using the double disk synergy test. Briefly, disks of ceftazidime, cefotaxime, aztreonam, and cefepime (30 μg each) were placed at a distance of 20 mm (center to center) from a disk containing amoxicillin (AMC, 20 μg) and clavulanic acid (CLA, 10 μg) on Mueller Hinton agar plates [2]. Phenotypic screening for carbapenemase production was performed by the modified Hodge-test with a meropenem disk, and by the imipenem-EDTA double-disk synergy test on Mueller Hinton agar plates [5].

### Detection of β-lactamase genes

DNA was extracted from the carbapenem-resistant *P. aeruginosa* using a Quick-gDNA MiniPrep kit (Zymo Research, Murphy, USA). *bla* genes were detected by polymerase chain reaction (PCR), using specific primers for genes encoding MBLs (*bla*_VIM_, *bla*_IMP_, *bla*_GIM_, *bla*_NDM_, *bla*_SPM_, *bla*_SIM_), KPC (*bla*_KPC_)_,_ ESBLs (*bla*_PER_, *bla*_VEB_, *bla*_SHV_, *bla*_TEM_, *bla*_CTX-M,_*bla*_GES_), and OXA type β-lactamases (*bla*_OXA-40,_*bla*_OXA-48,_*bla*_OXA-50_), as described previously [2,4,16]. The PCR products obtained from the VIM-positive isolates (n = 80) were sequenced on both DNA strands using an ABI3730 DNA sequencer (Applied Biosystems, Warrington, United Kingdom). Nucleotide and deduced protein sequences were identified by comparing the sequences with the database developed by Jacoby and Bush (http://www.lahey.org/Studies).

### Molecular typing of isolates

Molecular typing was performed by BOX-PCR, as described previously [17] for carbapenemase-producing *P. aeruginosa*. MLST was performed for 55 representative of the aforementioned isolates, including all isolates of the major BOX-patterns and one isolate from each of the remaining BOX-patterns, as described previously [14]. Sequences were obtained from both DNA strands, and allelic profiles were determined and assigned to sequence types (STs). Novel alleles and STs were submitted to the *P. aeruginosa* MLST website (http://pubmlst.org/paeruginosa/). Non-overlapping groups of related STs were identified using eBURST, with the default setting for the definition of groups (http://eburst.mlst.net) [18].

### PCR mapping of class I integrons

Combinations of specific primers for the conserved segments (5′CS and 3′CS) of class I integrons together with the *bla*_VIM_ primers were used for PCR mapping of class I integrons, as described previously [19]. Both DNA strands of the PCR products were sequenced using additional primers specific for the *bla*_PSE-1_*, bla*_OXA-10_, *arr-7*, *aacA4, aacA7, smr-2*, and *dfrb* genes [19,20], using an ABI3730 DNA sequencer (Applied Biosystems).

### Collection of clinical data

The medical records of patients infected with or colonized by carbapenemase-producing *P. aeruginosa* were reviewed retrospectively. Prior to obtaining the clinical information for the patients, approval was received from the Ethics Committee of the UHL, which is represented by the Infection Control Committee (permission number 1234). The patients were assumed to have acquired MDR *P. aeruginosa* prior to hospitalization when the microbe was isolated within 48 h of admission, while colonization with MDR *P. aeruginosa* was defined by the absence of relevant symptoms. Clinical information included age, duration of hospitalization, previous hospitalization history (history of transfer from another hospital, hospitalized departments, and wards), underlying diseases, clinical outcome, medical care exposures, and antimicrobial therapy.

## Results and discussion

Of the 568 *P. aeruginosa* isolates, 284 (50%) were identified as resistant to carbapenems (MICs for imipenem ≥16 mg/L). Of the 284 carbapenem-resistant isolates, 80 (28%) isolates were positive for carbapenemase production by phenotypic screening and were positive for *bla*_VIM_ by PCR. The remaining 204 carbapenem-resistant *P. aeruginosa* isolates were negative for carbapenemase production by phenotypic screening and negative for all ESBL and carbapenemase genes tested. Thus, the carbapenem resistance phenotype of the latter isolates may be attributed to other resistance mechanisms, such as porin loss, increased efflux, and AmpC overexpression.

All VIM producers exhibited MDR phenotypes. Apart from being resistant to imipenem and meropenem (MICs ≥16 mg/L), all VIM producers showed resistance to ticarcillin/clavulanate (MICs ≥128 mg/L) and piperacillin/tazobactam (MICs ≥128 mg/L), and intermediate-resistance or resistance to ceftazidime (MICs of 16 to ≥64 mg/L). In addition to being resistant to β-lactams, these isolates were also resistant to ciprofloxacin (MICs ≥4 mg/L), and excluding two isolates, to amikacin (MICs ≥64 mg/L), gentamicin (MICs ≥16 mg/L), tobramycin (MICs ≥16 mg/L), and netilmicin (MICs ≥32 mg/L). All VIM producers were susceptible to colistin (MICs ≤2 mg/L). The VIM producers showed higher MIC_50_ values for caftazidime (MICs ≥64 mg/L) and tobramycin (≥16 mg/L) compared with the non-VIM producing carbapenem-resistant *P. aeruginosa* (MICs of 4 mg/L and 2 mg/L, respectively). No other differences were observed for the MIC range, MIC_50_, and MIC_90_ values of the remaining antimicrobial agents tested.

**Figure 1:**
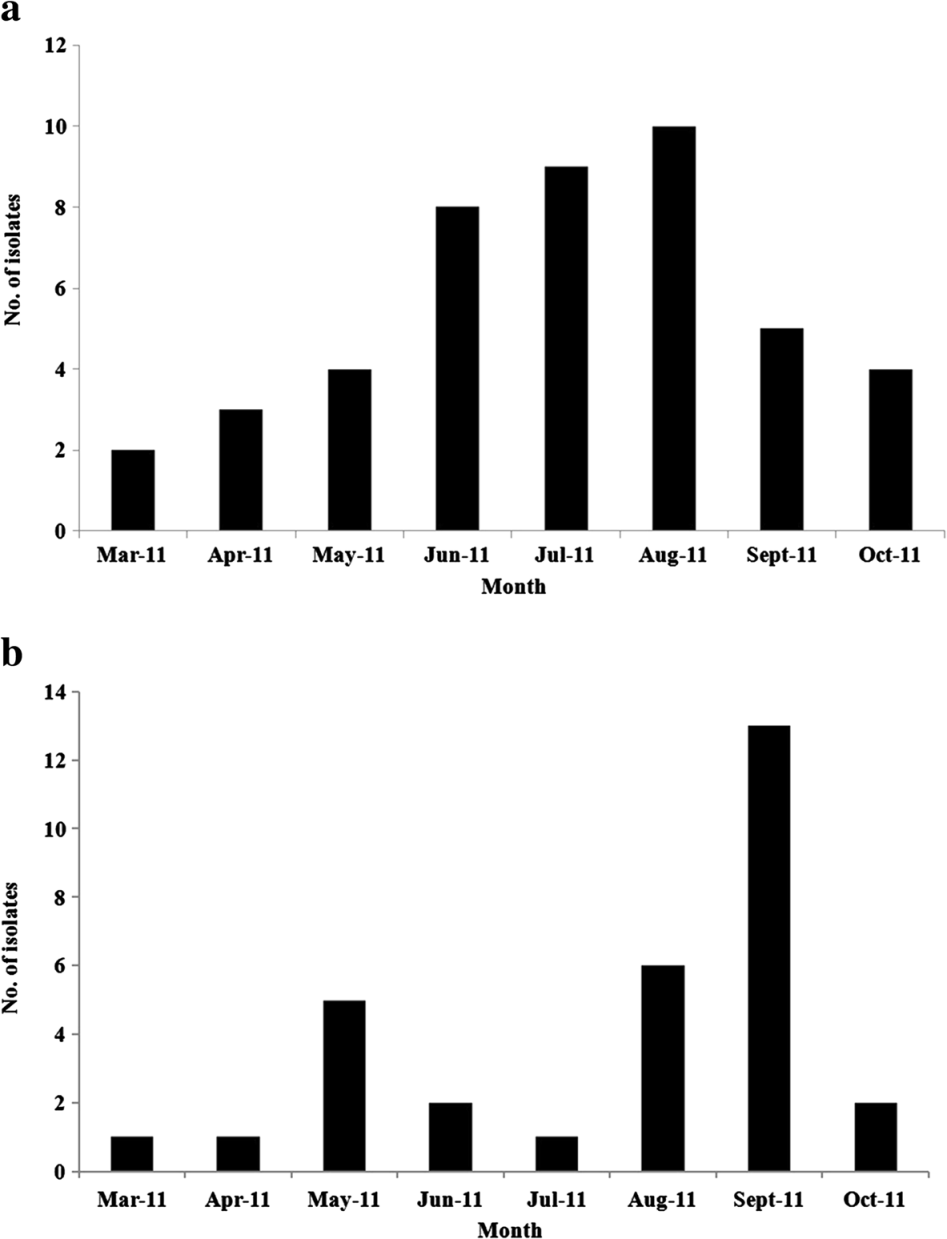
**Monthly distribution of a) VIM-2 and b) VIM-4 *P. aeruginosa *** **from March to October 2011.**

Of the 80 VIM producers, 48 encoded VIM-2, 31 had VIM-4, and one isolate contained VIM-17 enzymes. The monthly distribution of VIM-2 and VIM-4 producers is shown in Figure 1. An increase in the recovery rates of VIM-2 producers was observed from March to August 2011, whereas VIM-4 producers were recovered at higher frequencies in May and September 2011. The VIM producers were recovered from various wards of the hospitals, but mainly from the internal medicine (26%) and intensive care units (ICUs) (13%). Most of the VIM producers were isolated from urine (20.6%), but they were also recovered from sputum, blood, bronchial secretions (13% each), and other clinical specimens.

According to the clinical records, 52 of the 80 VIM-positive patients were infected, and 28 of 80 were colonized. A retrospective review of the clinical records of the patients infected with or colonized by VIM-producing *P. aeruginosa* was performed. All patients were diagnosed with an underlying disease at admission (such as solid tumors, diabetes, or cerebrovascular accidents), and 50% had a high fever. All patients had a previous hospitalization history (75% had been transferred from another hospital) and had received therapy with antimicrobial agents of multiple classes (e.g. penicillin-inhibitor combinations, second and third-generation cephalosporins, carbapenems, fluoroquinolones, glycopeptides, and cyclic lipopeptides). All but three cases of patients infected or colonized by VIM-producing *P. aeruginosa* were >65 years of age. The majority of infected patients were treated with a combination of colistin and aminoglycosides.

Two major BOX-PCR patterns were identified: P1 amongst VIM-2 and P2 amongst VIM-4 producers, which consisted of 21 (26%) and 16 (20%) out of the 80 VIM producers, respectively (Table 1). The remaining VIM producers were distributed into 18 different BOX-patterns, each comprised of one to five isolates. Amongst the 55 representative VIM-producers (all isolates of the major BOX-patterns and one isolate from each of the remaining BOX-patterns), we identified eight different STs. The international clones ST111 (clonal complex CC111) and ST235 (CC235) were the most prevalent, comprising 26 and 21 of the 55 VIM producers genotyped, respectively (Table 1). Both STs have been reported previously among VIM producers recovered from the university hospital of Patras, South Greece [21], and from other European countries, such as Sweden, Germany, France, Italy, Spain, and Bulgaria [12,13,21-26].

**Table 1:** **Gene cassettes, STs/CCs and BOX-patterns of the VIM-producing** ***P. aeruginosa*** **isolates**

**Gene cassettes**^**a**^	**GenBank; reference**	**No. of isolates**	**ST/CC**	**BOX pattern (no. of isolates)**
*aacA29a*|*bla*_VIM-2_ |*aacA29b*	EU118149; [20]	45	111/ CC111	P1 (21)
			111/ CC111	P5 (3)
			111/ CC111	P9 (4)
			111/ CC111	P11 (2)
			111/ CC111	P14 (1)
			111/ CC111	P16 (5)
			244/ CC244	P7 (2)
			244/ CC244	P15 (1)
			253/ CC253	P4 (1)
			277/ CC277	P6 (3)
			773	P8 (2)
*bla*_VIM-4_|*arr-7*|*aacA4*|*bla*_PSE-1_	FN397623; [19]	31	235/ CC235	P2 (16)
			235/ CC235	P17 (3)
			235/ CC235	P18 (3)
			235/ CC235	P19 (3)
			235/ CC235	P20 (4)
			235/ CC235	P10 (2)
*aacA29a*|*bla*_VIM-17_|*aacA29b*	EU118148; [20]	1	1457/ CC235	P3 (1)
*bla*_OXA-10_|*aacA4*|*bla*_VIM-2_|*smr-2*	KC527014; this study	2	395/ CC395	P12 (2)
*bla*_VIM-2_| *aacA7*|*dfrB*	KC527015; this study	1	235/ CC235	P13 (1)

^a^Aminoglycoside (6′) acetyltransferase genes: *aacA29a*, *aacA29b*, *aacA4, aacA7*; β-lactamase genes: *bla*_VIM-2_, *bla*_VIM-4_, *bla*_VIM-17_, *bla*_OXA-10_, *bla*_PSE-1;_ dihydrofolate reductase type B (trimethoprim resistance) gene: *dfrB*; ADP-ribosyl transferase (rifampicin resistance) gene: *arr-*7; small multidrug resistance protein gene: smr*-2*.

**Figure 2:**
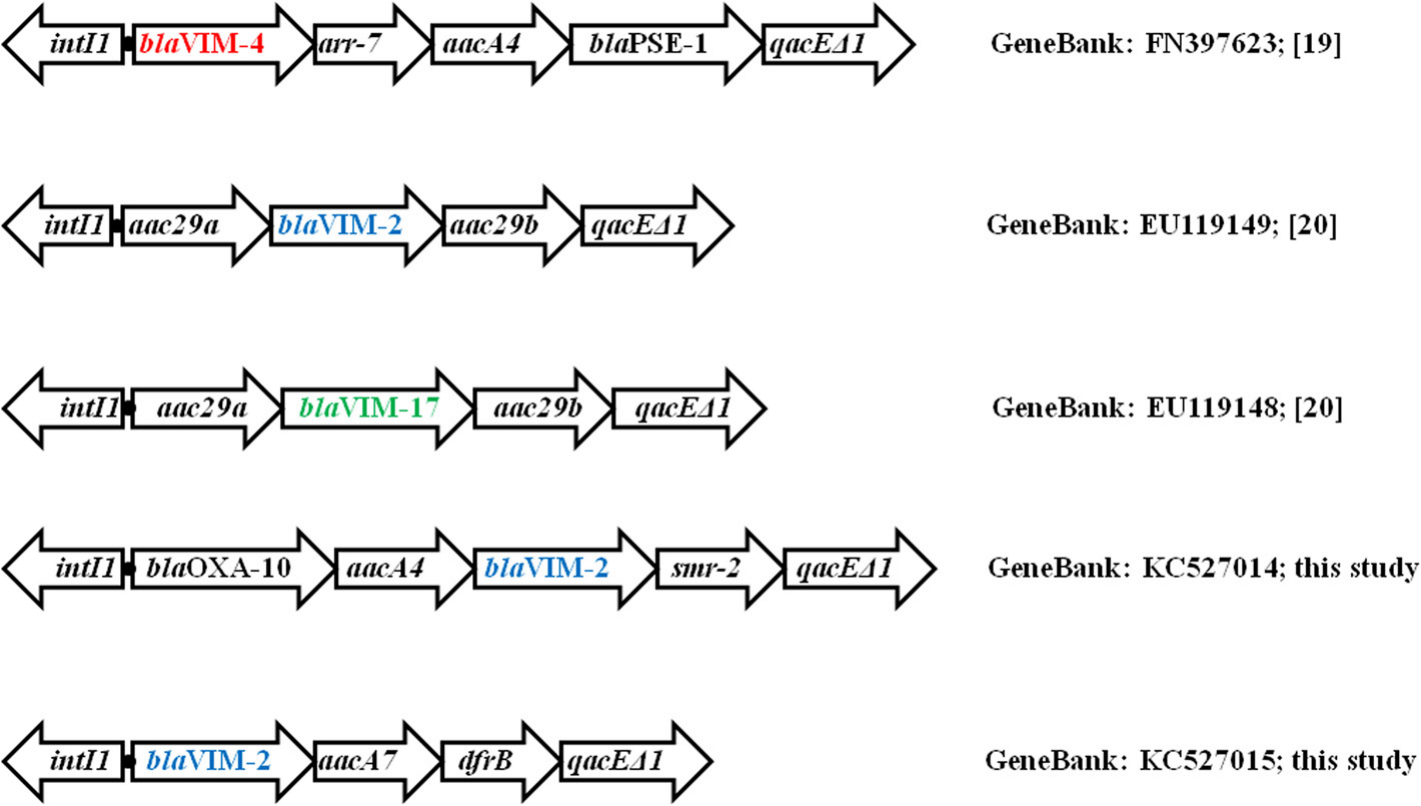
**Schematic representation (not to scale) of class I integrons of VIM-carrying *P. aeruginosa *isolates.** The GenBank accession numbers of the sequences are also indicated.

Five different class I integrons were identified: three structures coding for VIM-2 (*aacA29a*|*bla*_VIM-2_|*aacA29b, bla*_VIM-2_|*aacA7*|*dfrB,* and *bla*_OXA-10_|*aacA4*|*bla*_VIM-2_|*smr-2*), one for VIM-4 (*bla*_VIM-4_|*arr-7*|*aacA4*|*bla*_PSE-1_), and one for VIM-17 (*aacA29a*|*bla*_VIM-17_|*aacA29b*) (Table 1, Figure 2). Three of the five class I integrons found here have been described previously in *P. aeruginosa*[19,20], whereas in the remaining two the *bla*_VIM-2_ gene was found as part of novel gene cassette arrays [GenBank: KC527014 and KC527015].

In the current study, the *aacA29a*|*bla*_VIM-2_|*aacA29b* gene cassette array (In*59*-like) has been identified in 45 VIM-2 producers belonging to STs 111, 244, 253, 277, and 773. The majority of isolates assigned to ST111 were recovered from ICUs (15 out of 26 ST111 isolates; 58%), and they were isolated mainly from bronchial secretions and blood. These results were in concordance with other studies, where, respiratory care procedures among intubated patients in the ICU have been reported to account for the spread of MDR *P. aeruginosa*, and for the high mortality rates (40–60%) in bacterial nosocomial pneumonia and ventilator-associated pneumonia [27]. However, ST111 isolates were also found in other wards of our institutions; thus clonal expansion of VIM-2-producing ST111 isolates was documented. In addition, STs 244, 253, 277, and 773 carrying the In*59*-like cassette array were recovered from our internal medicine units. It is known that VIM-2 producers carrying the In*59*-like integron have previously been reported in Greece [20, GenBank: EU118149]. Single nucleotide variants in the 59-base element of *aacA29b* among VIM-producers have been described in Sweden, with one isolate originating from Greece [19, GenBank: AF263519]. The presence of the In*59*-like cassette in different strains/genetic backgrounds underscores the recruitment potential of this integron, and may indicate its association with a mobile structure.

The *bla*_VIM-4_|*arr-7*|*aacA4*|*bla*_PSE-1_ gene cassette array has been described in Scandinavia, where it was identified in two MBL-producers that were imported from Greece and Cyprus, one of which was also ST235 [20, GenBank: FN397623]. In the current study, all VIM-4 producers (n = 31) belonged to a single ST (ST235), and were isolated mainly from urine specimens (15 isolates, 50%) from the internal medicine (12 isolates) and urology units (ten isolates). However, VIM-4 producers were also isolated from other wards of the participant hospitals, suggesting clonal expansion of a single strain. From March to August 2011, VIM-4 producers of ST235 were isolated mainly in the internal medicine unit of UHL, whereas spread of these isolates in the urology unit was documented in September 2011.

The two novel VIM-2 encoding integrons (*bla*_VIM-2_|*aacA7*|*dfrB* and *bla*_OXA-10_|*aacA4*|*bla*_VIM-2_|*smr-2*) were found in isolates of STs 235 and 395 (Table 1). The *bla*_VIM-17_ (a variant of *bla*_VIM-2_) gene was found in a single isolate of a novel ST (ST1457), a single locus variant of ST235. *bla*_VIM-17_ was carried by an In*59-*like structure that has also been reported in *P. aeruginosa* from Greece [20, GenBank: EU118148].

## Conclusions

Multidrug-resistant VIM producers comprised 14.1% of the total *P. aeruginosa* recovered in Thessaly during 2011. Since the first report of VIM-producing *P. aeruginosa* in 2000 [28,29], this microorganism remains among the most prevalent MDR pathogens in Greece. VIM-1, VIM-2, VIM-4, and VIM-17-producing *P. aeruginosa* have been reported previously in Greece [21,28-30]. In the present study, the association between the genetic context and molecular types of VIM-producing *P. aeruginosa* was assessed. Most (46%) of the VIM producers belonged to the internationally-distributed clusters of ST111 and ST235, which carried previously-reported gene cassettes coding for the VIM-2 and VIM-4 enzymes, respectively. Novel VIM-2-carrying integron structures were identified among sporadic isolates. The VIM-17 carrying integron was found in an isolate of a novel ST (ST1457). The findings of this study underscore the high prevalence of VIM producers among carbapenem-resistant *P. aeruginosa* in Thessaly, as well as their ongoing evolution.

## Abbreviations

CC: Clonal complex; ESBL: Extended-spectrum β-lactamase; GES: Guiana extended-spectrum β-lactamase; IBC: Integron-associated class A β-lactamase; ICU: Intensive care unit; IMP: Imipenemase; KPC: *Klebsiella pneumoniae* carbapenemase; MBL: Metallo-β-lactamase; MDR: Multidrug-resistant; MIC: Minimum inhibitory concentration; MLST: Multilocus sequence typing; PCR: Polymerase chain reaction; ST: Sequence type; UHL: University Hospital of Larissa; VIM: Verona integron-encoded metallo-β-lactamase.

## Competing interests

The authors have no conflicts of interests to declare.

## Authors’ contributions

EP, AM, and VM conceived and designed the study. AL and AM performed the genetic analysis and molecular typing of isolates. AL and AT collected and reviewed the clinical data. AM was conducted the study, managed the data, and wrote the first draft of the paper. EP and VM interpreted the data. Other co-authors participated in data analysis, data interpretation, and contributed to the final draft. All authors read and approved the final manuscript.

## Pre-publication history

The pre-publication history for this paper can be accessed here:

http://www.biomedcentral.com/1471-2334/13/505/prepub
